# Degree of swallowing impairment in the elderly: clinical and instrumental assessment

**DOI:** 10.1016/j.bjorl.2024.101426

**Published:** 2024-03-25

**Authors:** Tatiane Totta Salgado, Cris Magna dos Santos Oliveira, Marina Gatti, Roberta Gonçalves da Silva, Heitor Marques Honório, Giédre Berretin-Felix

**Affiliations:** a Universidade de São Paulo, Faculdade de Odontologia de Bauru, Bauru, SP, Brazil; b Universidade Estadual Paulista Júlio de Mesquita Filho, Marília, SP, Brazil

**Keywords:** Aged, Deglutition, Endoscopy, Digestive system, Clinical protocols

## Abstract

**Objective:**

To classifying the degree of swallowing impairment in the elderly, comparing clinical and instrumental assessment.

**Methods:**

This is a cross-sectional study with quantitative and qualitative analysis of clinical and instrumental assessment of 37 elderly, aged 60–82 years, of both genders without neurological, oncological or systemic diseases, participated in this study. All participants were submitted to clinical evaluation and their results compared through fiberoptic endoscopic evaluation of swallowing considering liquid, pudding and solid food consistencies. Data were analyzed descriptively and statistically using the analysis of variance test (two-way ANOVA) and Tukey's *post hoc* test (*p* <  0.05).

**Results:**

In the clinical evaluation there was a higher occurrence of moderate swallowing impairment, followed by functional swallowing, while in fiberoptic endoscopic evaluation of swallowing the severity of the impairment was greater for moderate and mild degrees. There was no statistical difference between the clinical and instrumental evaluation methods. However, there was a significant interaction between the variables, with a difference for liquid consistency in the instrumental evaluation method.

**Conclusion:**

Healthy elderly have different degree of swallowing impairment according to food consistency. The clinical assessment using a scale that considers the physiological changes of the elderly, presented results similar to those found in the instrumental examination.

**Level of Evidence:**

2.

## Highlights

• Elderly presented moderate swallowing impairment for consistencies.
• There was no difference between clinical and instrumental evaluation.
• The assessment of swallowing in the elderly must consider physiological changes.

## Introduction

The aging process result in important impairments in the physiology of swallowing related to the oral, pharyngeal and esophageal phases,^1^, ^2^ resulting in difficulties in preparing, controlling and ingesting the food bolus and often leading to malnutrition, aspiration pneumonia and impairment on quality of life.^3^, ^4^

In the general elderly population, it can be observed the oral and pharyngeal phase of swallowing may be prolonged, decreased posterior and superior lingual movement, delayed triggering of the swallowing reflex, reduced hyolaryngeal movement and delayed opening of the upper esophageal sphincter. The reasons for these changes are diverse and may include decreased taste and olfactory acuity, dental problems, loss of muscle mass, sarcopenia and decreased tissue elasticity. These age-related modifications are usually compensated for and can be defined as prebisphagia. However, when compensation is not possible or in view of the association with diseases, the risk for the development of oropharyngeal dysphagia is increased.^5^, ^6^

According to the “World Population Prospects 2022” report released by the United Nations, the world population aged 65 or over is expected to increase from 10% to 16% in 2050. According to the report, the number of people considered elderly will be more than double the number of children under 5-years old.^7^

When present, dysphagia can be an important predictor for the development of serious complications in the elderly population, such as aspiration pneumonia, in addition to malnutrition and dehydration.^8^ This condition in this population may be associated with poor oral hygiene, reduced physical performance, changes in cognitive functions and also polypharmacy.^9^, ^10^ Taking into account that eating and drinking is part of social life, dysphagia can be associated with depression, social isolation and decreased quality of life.^11^

Considering the significant increase of the elderly population and the high prevalence of dysphagia in these individuals,^6^ the importance of screening, to identify elderly people at risk for dysphagia and those who are at risk of aspiration, aiming at the early diagnosis through clinical and instrumental evaluation of swallowing, is highlighted.^12^

To verify the evidence-based validity and effectiveness of clinical evaluation methods for oropharyngeal dysphagia, systematic reviews with studies involving patients with neurological disorders, were conducted.^13^, ^14^, ^15^ According to the analysis carried out in these studies, the water swallowing test was the most used.^13^, ^14^ Also, the water swallowing test, cough observation and vocal changes, was considered the best screening method.^13^ Besides this, only two relevant screening tests with acceptable sensitivity ≥70% and specificity ≥60%, namely the water swallowing test, as already mentioned, and the volume-viscosity swallow test, were identified.^15^

Thus, the divergence of the results found in the literature about the reliability of the clinical evaluation of swallowing to identify laryngeal penetration and laryngotracheal aspiration when compared to instrumental exams is clear, as well as the lack of a sensitive and reliable instrument aimed at the elderly population. In this sense, the objective of the present study is classifying the degree of swallowing impairment in the elderly, comparing clinical and instrumental assessment.

## Methods

This is a cross-sectional study with quantitative and qualitative analysis of clinical and instrumental assessment, with a factorial design considering a dependent variable degree of swallowing impairment and two independent variables: Evaluation method, being considered in two levels clinical evaluation and fiberoptic endoscopic evaluation and consistency of the food to be swallowed in 3 levels liquid, pudding and solid. This study was approved by the institution's Committee for Ethics in Research Protocol nº 111/2006. The recruited participants were part of a project, whose objective was to investigate voice, speech and orofacial functions of elderly people submitted to different oral prosthetic rehabilitation strategies. All participants signed the Informed Consent Form, agreeing to participate in the study.

Participants were 37 elderly people, 21 women and 16 men, aged between 60 and 82-years 58.83 ± 5.58, from the dental clinics of the institution where the study was conducted, as well as from private dental clinics and elderly members of the Association of Retirees and Pensioners of the municipality.

For the selection of the participants, the following inclusion and exclusion criteria were considered:

Inclusion – a) Good general health, represented by the absence of a history of neurological, oncological of the head and neck, psychological or psychiatric diseases; b) Adequate cognitive performance, assessed by the Mini-Mental State Examination^16^ score equal to or above 18 for illiterate individuals and equal to or above 24 points for literate individuals; c) Use of satisfactory, stable, adapted dental prostheses with adequate vertical dimension of occlusion, upon evaluation by a dentist.

Exclusion – a) No history of alcoholism and smoking; b) No use of medications that could cause xerostomia antidepressants, antispasmodics, bronchodilators, anticholinergics, antihistamines and sedatives.

### Clinical evaluation of swallowing and classification of the degree of swallowing impairment in the elderly

Swallowing was clinically evaluated by two experienced Speech-Language Pathologist and recorded, during the ingestion of three food consistencies liquid 10 mL of filtered water measured with a syringe and offered in a disposable cup, pudding 30 mL of filtered water added to 2 g of dietary grape juice powder thickened with a measure of instant thickener, offered with a disposable dessert spoon, compatible with 10 mL and solid a slice of bread roll 1 cm thick and approximately 4 cm in diameter, with a digital camera, for later analysis.

For the analysis, the parameters recommended by the literature,^17^, ^18^, ^19^ namely: anterior oral spillage, head movement, excessive participation of facial and cervical muscles, nasal reflux, alteration of cervical auscultation, respiratory alteration, cyanosis, vocal alteration, throat clearing, cough, alteration in the elevation of the larynx, residues in the oral cavity, increased oral phase time and multiple swallows^12^, ^20^, ^21^ four or more,^22^, ^23^ were used.

Regarding the time of the oral phase, the criterion of normality established in the literature,^24^ considering the three consistencies, where the average time intervals of 1.07 ± 0.35 s for the liquid consistency; 3.48 ± 1.76 s for the pudding consistency and 27.88 ± 7.11 s for the solid consistency, was used. Values higher than those stipulated were considered as indicative of increased oral phase time. It is noteworthy that to measure the time of the oral phase in the solid consistency, the times of chewing equivalent to the preparatory oral phase of swallowing and the oral phase itself were timed.^25^ As scales for classifying swallowing in the elderly are not found in the literature, the authors considered their own classification, constructed based on the observed clinical parameters observed, taking into account the physiological adaptations of swallowing in the elderly.

Classification of the degree of swallowing impairment according to clinical parameters.

**Table:** 

Classification	Clinical parameters
Normal Swallowing degree 0A	Absence of changes to any aspects evaluated for the consistencies tested.
Functional Swallowing degree 0B	Swallowing without signs of risk of penetration and aspiration, occurring up to 3 swallows, adequate oropharyngeal transit time, however, difficulties expected, such as: excessive participation of the facial and/or cervical muscles, head movement, with the absence of other factors.
Mild Swallowing Impairment degree I	Presence of the clinical signs described in degree 0B, which may also include anterior oral spillage, residues in the oral cavity, increased oropharyngeal transit time and absence of other factors.
Moderate Swallowing Impairment degree II	Presence of signs that suggest the presence of laryngeal penetration cough, throat clearing, abnormal cervical auscultation, alteration in vocal quality and alteration in laryngeal elevation, with the possibility of finding signs of degree I, in addition to nasal reflux and multiple swallows four or more swallows.
Severe Swallowing Impairment degree III	Presence of the signs described in degree II, with the possibility of presenting substantial food residues in the oral cavity, inefficient or absent cough, plus other signs that suggest the presence of aspiration, such as respiratory alteration and change in facial color.

### Fiberoptic endoscopic evaluation of swallowing

Fiberoptic endoscopic evaluation of swallowing FEES was performed by an Otorhinolaryngologist and directed by a Speech-Language Pathologist, blinded to the previous clinical evaluation. A flexible fiberoptic endoscopic equipment of the bronchoscope type and a nasopharyngoscope were used. The light source was used, and the images were captured via an image processor, and transmitted and recorded on a VCR. Topical anesthetic 2% xylocaine gel was applied over the circumference of the optical fiber, so that it would not cause discomfort during its introduction into the nasal cavity of the participant, however, direct anesthesia of the oropharynx to avoid consequent alteration in swallowing was not used.

During the examination, the elderly remained seated, with their heads positioned in the direction of the body axis, without flexion or rotation. Then, the optical fiber was introduced through one of the nostrils over the middle meatus, and subsequently deflected downwards and directed towards the oropharynx until the distance at which the entire pharyngeal and laryngeal region could be viewed panoramically. The fiber position was readjusted if the subject noticed any discomfort during swallowing.

First, the sensitivity test, with the touch of the distal extremity of the bronchoscope bilaterally in the region of the aryepiglottic ligaments, being considered adequate in the presence of the protective cough reflex, was applied.^22^, ^26^ Afterward, the device was positioned above the base of the tongue and the standardized foods, with the same consistency and quantity previously described in the clinical evaluation of swallowing, but artificially colored with blue food coloring, were offered. It is noteworthy that to offer the liquid consistency 10 mL a syringe was used.

The presence of the following aspects was observed for all consistencies offered: posterior oral spillage, delay in the beginning of the pharyngeal phase of swallowing, residues at the base of the tongue, residues in the valleculae and piriform sinuses, presence of cough, laryngeal penetration, laryngotracheal aspiration^27^ and the number of swallows, considering up to three swallows to be normal for complete pharyngeal clearance.^26^, ^28^

The data obtained by FEES were used to verify the occurrence of laryngeal penetration and laryngotracheal aspiration, that is, the severity of the functional impairment of swallowing. The classification, for comparison with the findings of the clinical evaluation of swallowing, was carried out according to the scale by Macedo Filho 2003:^23^

Normal – 0: normal oral containment, present reflexes, absence of salivary stasis, feeding, and aspiration, fewer than three attempts to propel for bolus clearance.

Mild – I: small post-swallow stasis, fewer than three propulsion attempts to clear the bolus, absence of nasal regurgitation and laryngeal penetration.

Moderate – II: moderate salivary stasis, increased post-swallow stasis, mre than three attempts to propel the bolus, nasal regurgitation, reduced laryngeal sensitivity with penetration into the laryngeal vestibule, but without laryotracheal aspiration.

Severe – III: significant salivary stasis, marked worsening of post-swallow residues, weak or absent propulsion, nasal regurgitation, tracheal aspiration.

### Statistical analysis

A descriptive analysis of the results of the clinical evaluation and FEES was performed. Aiming the comparisons between groups considering the two independent variables of this research Evaluation method and Food consistency, an analysis of variance model two-way ANOVA and Tukey's complementary test were performed, adopting a significance level of 5% *p* <  0.05.^29^

## Results

In the clinical evaluation of swallowing, considering all consistencies tested, there was a higher occurrence of moderate swallowing impairment, followed by functional swallowing. The presence of severe swallowing impairment was not verified. Likewise, in FEES, no occurrences of laryngotracheal aspiration or other signs of degree III were found Table 1.

**Table 1:** Classification of the degree of swallowing impairment in the clinical and fiberoptic endoscopic evaluation of swallowing, according to food consistencies.

Swallowing evaluation	Food consistencies		
	Liquid	Pudding	Solid
Scale of Degree of swallowing impairment			
0A – Normal Swallowing	4 (10.81)	4 (10.81%)	1 (02.70%)
0B – Functional Swallowing	11 (29.73%)	14 (37.84%)	8 (21.92%)
I – Mild Swallowing Impairment	7 (18.92%)	5 (13.51%)	10 (24.32%)
II – Moderate Swallowing Impairment	15 (40.54%)	14 (37.84%)	18 (48.65%)
III – Severe Swallowing Impairment	0 (0.00%)	0 (0.00%)	0 (0.00%)
Fiberoptic endoscopic evaluation of swallowing (FEES)^22^			
0 – Normal	18 (48.64%)	6 (16.21%)	3 (8.10%)
I – Mild	13 (35.13%)	15(40.54%)	12(32.43%)
II – Moderate	6 (16.21%)	16 (43.24%)	22 (59.45%)
III – Severe	0 (0%)	0 (0%)	0 (0%)

FEES findings showed greater swallowing impairment for moderate and mild degree, respectively. Furthermore, the occurrence of impairments in the oral phase of swallowing for all consistencies, mainly for the posterior oral spillage and residues at the base of the tongue aspects, in addition to impairments also in the pharyngeal phase, such as the delay in the beginning of the pharyngeal phase, residues in valleculae and piriform sinuses, is also highlighted Table 2.

**Table 2:** Frequency of findings of fiberoptic endoscopic evaluation of swallowing for liquid, pudding and solid consistencies.

Fiberoptic endoscopic evaluation of swallowing (FEES)^22^	Food consistencies		
	Liquid	Pudding	Solid
Main findings			
Posterior oral spillage	10 (27.03%)	22 (59.46%)	26 (70.27%)
Delay in the beginning of the pharyngeal phase of swallowing	10 (27.03%)	22(59.46%)	26(70.27%)
Residues at the base of the tongue	1 (2.70%)	8 (21.62%)	25 (67.57%)
Residues in valleculae	9 (24.32%)	9 (24.32%)	23 (62.16%)
Retention in piriform sinuses	7 (18.92%)	3 (8.11%)	14 (37.84%)
Cough	4 (10.81%)	2 (5.41%)	3(8.11%)
Laryngeal penetration	4 (10.81%)	2 (5.41%)	3(8.11%)
Laryngotracheal aspiration	0 (0.00%)	0 (0.00%)	0 (0.00%)

Regarding the comparisons performed by Two-way ANOVA, the means and standard deviations for each consistency in the clinical evaluation and FEES are presented in Table 3. There was no significant difference between the scale classification degree by clinical evaluation scale and instrumental evaluation FEES *p* >  0.05. However, there was a significant interaction between both independent variables: consistencies and evaluation method *p* =  0.01. In this case, for FEES, the values for liquid consistency were significantly lower 0.61 ± 0.69 when compared to pudding 1.27 ± 0.75 and solid 1.51 ± 0.65 consistencies, which, in turn, demonstrated greater severity.

**Table 3:** Comparison of the degree of swallowing impairment between the clinical and instrumental evaluation (FEES) for liquid, pudding and solid consistencies.

Food Consistencies	Evaluation	Swallowing impairment
		Median ± SD
Liquid	Clinical	1.00 ± 0.91^a,b^
	FEES	0.61 ± 0.69^c^
Pudding	Clinical	0.89 ± 0.93^a,c^
	FEES	1.27 ± 0.75^a,b^
Solid	Clinical	1.29 ± 0.81^a,b^
	FEES	1.51 ± 0.65^b^

FEES, Fiberoptic Endoscopic Evaluation of Swallowing.

Two-way ANOVA and Tukey’s test (*p* <  0.05).

*Distinct superscript letters represent a statistically significant difference.

## Discussion

The prevalence of dysphagia increases with age, even the changes caused by the natural aging process can lead to swallowing difficulties that contribute to undesirable outcomes, such as decreased food intake, malnutrition and dehydration.^30^, ^31^ In this context, swallowing assessment stands out, considering early identification and necessary adaptations.

The clinical evaluation of swallowing showed a predominance of moderate swallowing impairment in this population, with occurrence of cough and vocal alteration. As the literature points out, in a study with post stroke patients, the occurrence of at least two signs of risk indicators for dysphagia, such as cough and voice impaiments after swallowing, can differentiate moderate to severe swallowing impairment from a mild swallowing impairment or normal swallowing, allowing to select which patients need instrumental evaluation.^32^

Functional swallowing was the second most frequent classification, and the presence of severe swallowing impairment was not verified. Such findings were expected, considering that the sample involved healthy elderly people.

In FEES, impairments in the oral and pharyngeal phases of swallowing for all consistencies, such as the occurrence of posterior oral spillage, delay in the beginning of the pharyngeal phase, residues at the base of the tongue, residues in valleculae, residues in piriform sinuses, cough and laryngotracheal penetration, in addition to the occurrence of multiple swallows, mainly in pudding and solid consistencies, were verified. These findings are in line with the literature^22^, ^33^, ^34^, ^35^, ^36^ and show that even elderly people who have natural teeth demonstrate a deteriorated physiological function of swallowing, such as changes in the oral and pharyngeal phases, generating multiple swallows, posterior oral spillage, oral and pharyngeal residues and laryngeal penetration.^36^, ^37^

Although the literature indicates a connection between increasing age and a higher prevalence of pharyngeal alterations in the elderly, such as pharyngeal stasis, delay in closing the laryngeal vestibule, laryngeal penetration and laryngotracheal aspiration,^36^ it is important to consider that, for the three consistencies offered, the clinical examination did not verify the presence of severe swallowing impairment degree III, in line with FEES findings in the sample of elderly people studied.

Considering the absence of non-parametric tests for qualitative data analysis, the parametric test for analysis of variance two-way ANOVA was used in the present study. As already demonstrated more than two decades ago, parametric tests on these occasions are easy to apply and can provide information as valuable as non-parametric ones.^29^

The comparison between the findings in clinical assessment, using the Classification of the Degree of Swallowing Impairment and those of FEES, for the three consistencies offered, showed that there was no significant difference. Through the descriptive analysis, the FEES findings showed a low prevalence of subjects with laryngeal penetration and a tendency of the clinical examination to overestimate the occurrence of laryngeal penetration, detected in the classification as moderate swallowing impairment, compared to FEES. A study carried out in 2016, which also used FEES, compared the clinical with the instrumental evaluation in individuals affected by stroke. In the study, the authors reveal that there is a relationship between the evaluation modalities, however, the clinical evaluation can lead to false positives and/or false negatives, being ideal the combination of both instruments, whenever possible.^38^

It is also necessary to take into account the consistency tested and the evaluation method. As demonstrated in the present study, when assessing swallowing with pudding and solid foods, there was greater severity in the instrumental examination compared to the clinical evaluation, with a greater predominance of premature posterior spillage and delay in the beginning of the pharyngeal phase. The literature points out that the most frequent alterations in the elderly population are precisely the alterations in the oral and pharyngeal phases.^39^, ^40^ The structural modifications of the oral cavity, associated with the impairment of the dentition or the use of inadequate dental prostheses and the reduction of tongue movements, may explain difficulties in the management of the food bolus culminating in premature escape and the presence of food residues in the oral cavity.^41^, ^42^ In addition, changes in the pharyngeal phase, such as delayed triggering of the swallowing reflex, decreased hyolaryngeal movement and increased presence of residues in the pharyngeal region, configuring a picture of presbyphagia.^5^, ^42^

Pudding and solid consistencies are directly affected by these alterations, as they require coordinated preparation and management of the food bolus. According to the results presented, another study found alterations in the oral phase of swallowing in 88% of its sample, with emphasis on greater involvement with solids, with the presence of residues in the oral cavity.^43^

In addition, regarding the pudding consistency, studies describe that thickened or naturally thicker foods can compromise the oral and pharyngeal phase of swallowing, since these foods require a certain force to propel the food bolus. Thus, the risk, especially in the elderly population, of the presence of residues in the oral cavity and pharynx increases.^44^

Some variables of this study should be considered for a better deepening of the issues studied here in the future, namely, the way of delivering food to the oral cavity for liquid consistency use of a cup in the clinic and a syringe in FEES; as well as the position of the head during the evaluations, considering that in the clinical evaluation the individual carried the liquid content stored in a glass to their mouth and, consequently, performed a cervical extension movement to swallow it, which could generate changes in the pharyngeal phase, since changes in posture can alter the speed and direction of the food bolus.^22^

Finally, it is important reflect on the development of instruments for the classification of swallowing impairment in the elderly population, that consider the physiological changes in swallowing in the elderly. With the increase in the elderly population, it will also be necessary to structure processes that can identify and separate presbyphagia and dysphagia.

## Conclusion

Healthy elderly have different degree of swallowing impairment according to food consistency. The clinical assessment using a scale that considers the physiological changes of the elderly, presented results similar to those found in the instrumental examination.

## Funding

Fundação de Amparo à Pesquisa do Estado de São Paulo (10.13039/501100001807FAPESP), process n° 06/60336-9.

## Conflicts of interest

The authors declare no conflicts of interest.
